# Consensus statement of the Italian society of pediatric allergy and immunology for the pragmatic management of children and adolescents with allergic or immunological diseases during the COVID-19 pandemic

**DOI:** 10.1186/s13052-020-00843-2

**Published:** 2020-06-16

**Authors:** Fabio Cardinale, Giorgio Ciprandi, Salvatore Barberi, Roberto Bernardini, Carlo Caffarelli, Mauro Calvani, Giovanni Cavagni, Elena Galli, Domenico Minasi, Michele Miraglia del Giudice, Viviana Moschese, Elio Novembre, Francesco Paravati, Diego G. Peroni, Maria Angela Tosca, Giovanni Traina, Salvatore Tripodi, Gian Luigi Marseglia, and the SIAIP Task Force, Doriana Amato, Caterina Anania, Elisa Anastasio, Rachele Antignani, Stefania Arasi, Martire Baldassarre, Ermanno Baldo, Andrea Barbalace, Simona Barni, Federica Betti, Annamaria Bianchi, Ezio Bolzacchini, Maira Bonini, Paolo Bottau, Sara Bozzetto, Maria Antonia Brighetti, Davide Caimmi, Silvia Caimmi, Luigi Calzone, Caterina Cancrini, Lucia Caminiti, Giulia Capata, Lucetta Capra, Carlo Capristo, Elena Carboni, Francesco Carella, Riccardo Castagnoli, Elena Chiappini, Fernanda Chiera, Iolanda Chinellato, Loredana Chini, Francesca Cipriani, Flavio Civitelli, Pasquale Comberiati, Daniele Contini, Stefania Corrente, Claudio Cravidi, Giuseppe Crisafulli, Barbara Cuomo, Enza D’Auria, Sofia D’Elios, Fabio Decimo, Auro Della Giustina, Rosa Maria Delle Piane, Maria De Filippo, Valentina De Vittori, Lucia Diaferio, Maria Elisa Di Cicco, Dora Di Mauro, Marzia Duse, Silvia Federici, Giuseppe Felice, Maria Grazia Fenu, Giuliana Ferrante, Tiziana Foti, Fabrizio Franceschini, Daniele Ghiglioni, Giuliana Giardino, Mattia Giovannini, Giovanni Cosimo Indirli, Cristiana Indolfi, Massimo Landi, Francesco La Torre, Lucia Maddalena Leone, Amelia Licari, Lucia Liotti, Vassilios Lougaris, Nunzia Maiello, Paride Mantecca, Sara Manti, Marco Maria Mariani, Alberto Martelli, Carla Mastrorilli, Violetta Mastrorilli, Davide Montin, Francesca Mori, Roberta Olcese, Giorgio Ottaviano, Claudia Paglialunga, Giovanni Pajno, Giuseppe Parisi, Stefano Pattini, Luca Pecoraro, Umberto Pelosi, Claudio Pignata, Giampaolo Ricci, Silvia Ricci, Stefano Rizzi, Caterina Rizzo, Sara Rosati, Paolo Rosso, Maria Sangerardi, Angelica Santoro, Francesca Saretta, Lucrezia Sarti, Marco Sartorio, Majla Sgruletti, Annarosa Soresina, Ifigenia Sfika, Mayla Sgrulletti, Nuccia Tesse, Valentina Tranchino, Alessandro Travaglini, Malizia Velia, Elvira Verduci, Mario Vernich, Elisabetta Veronelli, Stefano Volpi, Martina Votto, Anna Maria Zicari

**Affiliations:** 1 Pediatric Unit, Azienda Ospedaliero-Universitaria “Policlinico- Giovanni XXIII, Bari, Italy; 2 Allergy Clinic, Casa di Cura Villa Montallegro, Genoa, Italy; 3 Pediatric Unit, ASST-Rhodense, Rho, MI Italy; 4 Pediatric Unit, Empoli Hospital, Empoli, FI Italy; 5 Pediatric Clinic, Mother-child Department, University of Parma, Parma, Italy; 6 Operative Unit of Pediatrics, S. Camillo-Forlanini Hospital, Rome, Italy; 7 Coordinator European Allergology Center - European Diagnostic Center Dalla Rosa Prati, Parma, Italy; 8 Pediatric Allergology Unit, Department of Pediatric Medicine, S. Pietro Hospital Fatebenefratelli, Rome, Italy; 9 Pediatric Unit, Grande Ospedale Metropolitano, Reggio Calabria, Italy; 10 Department of Woman and Child and General and Specialized Surgery, University of Campania Luigi Vanvitelli, Naples, Italy; 11 Pediatric Allergology and Immunology Unit, University of Rome Tor Vergata, Policlinico Tor Vergata, Rome, Italy; 12 Allergy Unit, Department of Science Health, Meyer Children’s Hospital, University of Florence, Florence, Italy; 13 Pediatric Unit, Crotone Hospital, Crotone, Italy; 14 Pediatric Clinic, University of Pisa, Pisa, Italy; 15 Allergy Center, Istituto Giannina Gaslini, Genoa, Italy; 16 Pediatric Unit, ASST-Martesana, Milan, Italy; 17 Allergology Service, Policlinico Casilino, Rome, Italy; 18 Pediatric Clinic, Pediatrics Department, Policlinico San Matteo, University of Pavia, Pavia, Italy

**Keywords:** COVID-19, Pandemic, Child, Adolescent, Allergy, Asthma, Immunologic disease

## Abstract

The COVID-19 pandemic has surprised the entire population. The world has had to face an unprecedented pandemic. Only, Spanish flu had similar disastrous consequences. As a result, drastic measures (lockdown) have been adopted worldwide. Healthcare service has been overwhelmed by the extraordinary influx of patients, often requiring high intensity of care. Mortality has been associated with severe comorbidities, including chronic diseases. Patients with frailty were, therefore, the victim of the SARS-COV-2 infection. Allergy and asthma are the most prevalent chronic disorders in children and adolescents, so they need careful attention and, if necessary, an adaptation of their regular treatment plans. Fortunately, at present, young people are less suffering from COVID-19, both as incidence and severity. However, any age, including infancy, could be affected by the pandemic.

Based on this background, the Italian Society of Pediatric Allergy and Immunology has felt it necessary to provide a Consensus Statement. This expert panel consensus document offers a rationale to help guide decision-making in the management of children and adolescents with allergic or immunologic diseases.

## Introduction

A novel coronavirus, SARS-COV-2, lead to coronavirus disease 2019 (COVID-19). COVID-19 burst in China and rapidly spread worldwide. Italy was the first European country to be interested in the pandemic. South Lombardy was the first cluster, then, COVID-19 disseminated across Italy.

COVID-19 had an impressive impact on Medicine so that COVID-19 Medicine is a new term to define this topic. Thousands of papers are publishing, so the scientific community is addressed to the peculiar aspects of this infection. COVID-19 has pleiomorphic characteristics of presentation and severity. In particular, it has been reported that severe and lethal disease is associated with male gender, old age, and comorbidity. Fortunately, childhood seems to be preserved by severe COVID-19, and relatively few cases occurred still now. Every age may be affected, including infancy.

As chronic diseases have been associated with more severe COVID-19, the need to define pragmatic recommendations has emerged. Therefore, the executive board of the Italian Society of Pediatric Allergy and Immunology (SIAIP) has considered appropriate to disseminate a document including a series of recommendations for the management of allergies and immunological diseases in children and adolescents. All SIAP Committees have provided Consensus Statements. The current document is oriented to physicians and caregivers involved in the care of children and adolescents with the most common allergic and immunologic disorders. The literature search considered a time frame starting from 2020 January up to the end of April. The recommendations are mainly based on principles as very few primary data are available at present.

### Allergic rhinitis

In the current state of knowledge, topical nasal corticosteroid therapy for allergic rhinitis in children and adolescents with COVID-19 can be continued at the recommended posology [1, 2].

It is considered appropriate to continue treatment with antihistamine drugs regularly so as not to lose control of oculorhinitis symptoms in the seasonal period or due to the increased exposure to indoor allergens.

The interruption of topical nasal corticosteroids is not recommended, which does not seem to reduce the immune system. However, indeed the non-administration may lead to an increase in nasal respiratory symptoms, in particular, nasal obstruction with a more probable occurrence of potentially infected secretions and with a higher risk of bacterial colonization also of the lower airways. It should also be remembered that the increase in rhinitis symptoms with frequent sneezing leads to a higher potential spread of the virus. Moreover, as itching is a typical symptom of both allergic rhinitis and conjunctivitis, appropriate management of this symptom should be performed. Nose and eyes scratching is a relevant source of SARS-CoV-2 infection. Second-generation antihistamines should be, therefore, used to control nasal and ocular itching. Effective and safe oral medications should be preferred, such as well-proven molecules, such as cetirizine, loratadine, and fexofenadine, to relieve nasal and ocular complaints [3–5].

These recommendations have to be updated regularly in light of the continuous acquisitions on COVID-19.

### Asthma

Continue to administer medications prescribed to maintain asthma control regularly, in particular, inhaled corticosteroids (ICS), long-acting bronchodilators, antileukotrienic drugs, and, if necessary, oral corticosteroids (OCS) [2]. The suspension of the treatment can lead to a condition of poor or lack of control of the symptoms, which exposes more the child or adolescent to the risk of even severe asthma exacerbations. For patients with severe asthma, it is advisable to continue therapy with biological drugs and evaluate the possibility of home administration (or at a local hospital center). The only exception could be the suspension of biologics during the acute phase of COVID-19 infection.

Patients with asthma (particularly severe or uncontrolled asthma) are at increased risk of developing more severe COVID-19 [5–8]. Preexisting allergies have not been classified as a risk factor. However, Pediatric allergists must obtain the best control of asthma and the allergic condition and educate patients and their parents on current recommendations to reduce the risk of COVID-19. In particular, uncontrolled asthma is the most crucial risk factor for severe COVID-19 disease, so gaining and maintaining control is the clinical priority, regularly continuing with therapy according to the Guidelines [6–9].

Ensure that all patients have a written action plan with instructions on how to increase the dosage of the controller drug (“step-up”) and the use of bronchodilator drugs and OCS, in case of reappearance or worsening of symptoms. If possible, avoid the use of nebulizers (nebulizers increase the risk of spreading the virus to other subjects and health professionals) and prefer the administration of bronchodilators and ICS through a metered dosed inhaler (MDI) with the help of a spacer. Ensure patients or their parents know when to contact their doctor and/or medical Emergency helpline or go to the Emergency Room for acute worsening of asthma. Always prescribe asthma medications in more significant quantities to make up for the lack of supplies.

It is crucial for the clinicians, in the asthma management, to communicate the asthma action plan to patients and their parents, to obtain the maximum of adherence and the “patient engagement” in the regular treatment and the asthma exacerbations [2, 6–9].

Avoid spirometry in patients with confirmed/suspected infection by COVID-19, since the execution of this test can spread the virus and expose all staff and other patients to the risk of infection. Therefore, postpone the respiratory function tests or perform them only in case of real necessity if the patients and caregivers do not present flu-like symptoms (fever, cough, and dyspnoea) carrying out the maneuvers in a condition of maximum control of a possible contagion (disposable filter, instrument sterilization, PPE equipment). Follow the measures of social distancing and personal hygiene, washing the hands thoroughly, always wearing PPE, as part of the health activity and rigorous infection control procedures if aerosol generation procedures are required (for example nebulization, oxygen therapy, also with nasal cannulas, manual ventilation, non-invasive ventilation, and intubation).

In pandemic time, it is essential to avoid unnecessary risks with maneuvers such as the measure of lung function that can contribute to the spread of the virus to all healthcare workers and other patients [2, 6–9]. It is fundamental to strictly follow the measures of social distancing and preventive rules, especially when evaluating patients with respiratory symptoms, always ensuring cleanliness and hygiene of the sanitary tools used during the visit or other procedures [2, 6–9].

Inform patients and their parents and all those who have contact with the child to wash their hands and clean devices such as face masks, mouthpieces, spacers using an appropriate detergent following the manufacturer’s instructions and inform patients or their parents not to share inhalers, pre-dosed sprays and other devices with other subjects.

In the pandemic time, it is crucial for the clinicians, the patients, and parents to know the recommendations relating to prevention and sterility rules to avoid the spread of COVID 19 [2, 6–9].

It is recommended to avoid smoking for adults and adolescents, in particular at home (passive smoking for children).

Cigarette smoke, always and in any case harmful, causes an increase in the expression of ACE2 receptors, present mainly in the lower respiratory tract [10–13]. ACE2 is the coronavirus receptor: through binding with it, coronavirus penetrates the pulmonary alveolus and thus infects the individual. Therefore, an increased expression of ACE2, induced by smoking, means increased susceptibility to contract the COVID-19 infection and potentially to develop a more severe form.

### Food allergy

Consider the choice of “safe” food as a priority. Patients should always have an action plan with the updated dosage of necessary drugs (antihistamine, corticosteroid, and adrenaline) and two available adrenaline auto-injectors.

In children with food allergies (FA), the avoidance of the culprit food currently represents the primary treatment. The choice of “safe” food is crucial for allergic children and their families [2, 13]. However, with the advent of the pandemic, people may have difficulty accessing specialist allergy products, because of high demand in shops or low income due to the pandemic itself. In fact, in the pandemic era, drastic measures should be decided to limit access to healthcare resources [14]. However, no clear rationale still exists.

Thus, it can become challenging to find safe products for children allergic to one or more foods. The difficulty in finding products already consumed and tolerated can have an unwanted double effect: a) risk of buying new, unknown products that may be unsafe; b) risk of not correctly reading the labels of the products purchased. These effects increase the potential risk of exposure to the allergen and any anaphylactic reactions. Therefore, children with FA should always have an action plan with the updated dosage of necessary drugs (antihistamine, corticosteroid, and adrenaline) and two available adrenaline auto-injectors.

The child/adolescent suffering from food allergies must consume a well-balanced and varied diet, also rich in micronutrients. An adequate supply of these nutrients may be necessary. Calcium and vitamin D might be advisable in children with cow’s milk protein allergy, and when prolonged periods of reduction of sunlight exposure occur.

In children with food allergies, the exclusion diet is currently the primary treatment. Depending on the age of the child and the food/foods that are eliminated, nutritional deficiencies may arise [2, 13]. Specific micronutrients, including vitamins A, D, C, E, B6, and B12, folate, zinc, iron, copper, and selenium, have a synergistic role in the immune response [3]. Malnutrition can compromise the proper functioning of the immune system [2, 13–17]. Several studies highlight the role of micronutrients (trace elements, minerals, and vitamins) in the immunological mechanisms involved in response to infections. Therefore, recent studies recommend their regular intake, especially in preschoolers. The diet should be varied and complete to assure the right intake of macro and micronutrients. In particular, supplementation of Vitamin D at a dose of 600–1000 IU/day during meals would be advisable in this pandemic period that requires staying at home with a consequent reduction in exposure to sunlight. More generous dosages would be appropriate in the presence of risk factors, such as obesity, anti-epileptic therapy, and dark skin.

Oral food challenge (OFC) should be re-scheduled except for particular conditions. Outpatient visits of children affected by suspected FA, except for those with cow’s milk protein allergy who do not have a milk substitute or comorbidities, should be postponed.

When children with FA need milk formulas introduction (soy formula, rice milk, hydrolysate formula) under medical supervision, the oral food challenge (OFC) should not be deferred [1, 13]. A challenge should also be considered when a child is suspected of not tolerating the substitutive milk or is highly suspected of an allergy misdiagnosis resulting in an unnecessary elimination diet. In other situations (such as challenges to confirm presumed cross-reactivity or outgrown IgE-mediated FA), OFC should be re-scheduled. In the case of uncertain symptomatic ingestion, the reintroducing of food may be delayed, and telehealth services are an excellent alternative to face-to-face visits to evaluate these patients. Control visits at the office should be postponed unless severe systemic reactions suggesting a new food allergy is reported. Any new patient visit for an elimination diet trial may be delayed unless an IgE-mediated reaction, FPIES, or eosinophilic esophagitis are suspected. Priority should be given to patients suffering from recurrent idiopathic anaphylaxis.

In children with FA, initiation of oral Immunotherapy (OIT) should be postponed. If a patient is undergoing OIT, we suggest keeping the same daily dose of allergen at home until new medical indications.

Patients affected by some kind of food allergy (e.g., peanut allergy) may benefit from oral immunotherapy (OIT) [18]. OIT requires many in-hospital visits for a gradual increase of allergen dose ingestion. Allergen dose increases should be carried out under medical supervision because of the risk of potentially severe systemic reactions. Therefore, the initiation of OIT should be postponed. If a patient is undergoing OIT, we suggest maintaining the same daily dose of allergen at home until new medical indications are given.

### Atopic dermatitis

The presence of atopic dermatitis requires special precautions for the application of the necessary preventive hygiene measures (handwashing, face mask, and environmental hygiene) to avoid the onset of irritative/contact dermatitis.

The application of the hygiene rules recommended by the World Health Organization (WHO) can be difficult in the presence of eczema, due to the possible appearance of irritative and allergic contact phenomena. To minimize this risk, subjects suffering from atopic dermatitis should prefer to wash their hands with soap and water rather than with antiseptic agents (i.e., chlorhexidine) or alcoholic sanitizer, avoid excessive washing or with too hot water, dry well without rubbing and apply the moisturizer after washing and during the day. In the case of facial dermatitis, it is useful to apply a moisturizer/barrier cream before wearing a face mask, and avoid masks with metal wires in case of nickel allergy. During house cleaning, the use of nitrile gloves and a proper rinse of the contact surfaces may be useful. In case of worsening or suspicion of superinfection, it is advisable to contact the family doctor for further therapies [19–23].

The presence of atopic dermatitis or active skin lesions has not been associated with a higher risk of contracting SARS-CoV-2 infection.

AD is a syndrome characterized by immune dysregulation, but there is no evidence at present, nor any clinical reporting, which suggests that affected people are more likely to develop COVID-19 and/or to experience a more severe form [24, 25]. It is not currently known whether the transmission of the SARS-CoV-2 infection is more likely to occur in subjects with active or exuding skin lesions. However, as coronavirus appears to spread through respiratory droplets, it appears highly unlikely that a damaged skin barrier increases the risk of developing COVID-19.

Patients suffering from atopic dermatitis who are taking an immunosuppressant drug/biological drug/oral steroids must discontinue medication only on advice from their attending physician.

On April 9, 2020, the National Institute for Health and Care Excellence (NICE) published a series of recommendations to be adopted during the current COVID-19 pandemic for patients with skin diseases, including eczema, who carry out immunomodulatory therapies [26]. There is no data that Dupilumab can somehow increase the risk of SARS-CoV-2 infection, and it can also be done subcutaneously at home. Starting a new therapy with Dupilumab should be considered with extreme caution, while it is advisable to continue an ongoing therapy [27, 28] regularly. The British Association of Dermatologists has provided a grid for healthcare professionals to identify patients for whom COVID-19 poses a higher risk due to their disease and treatment. Interruption of drug therapy should only be done on the advice of the attending physician, who decide on an individual basis [29].

### Drug and latex allergy

It is recommended to delay the workup for suspected drug hypersensitivity if there is a well-tolerated drug with the same indication. The drug challenge can be done when there is an urgent need for a drug, and there is no alternative molecule.

The list of drugs that could be necessary to investigate in children for presumed hypersensitivity includes more common antibiotics and antipyretics, but also chemotherapeutics, perioperative drugs, and biological drugs. Drug provocation tests should always be done by trained personnel and in a setting with facilities to treat adverse reactions [30]. Physicians and nurses should not those involved in the treatment of patients with COVID-19. If there is a drug, supposed to be not cross-reactive, having the same indication of the suspected drug, which has been tolerated from the patient, the drug hypersensitivity workup could be delayed. The drug challenge should be done if there is an urgent and real need for a specific molecule (e.g., biological drugs or anti-cancer therapies) and no alternative for treatment and the best outcome.

So far, hydroxychloroquine, azithromycin, heparin, remdesivir, and biologic drugs (anti-IL-6 receptor and anti-IL-1 monoclonal antibodies) have been suggested as potential treatments for COVID-19 infection. In the case of suspected hypersensitivity to these drugs, patients should undergo a complete allergy workup to identify a safe alternative, considering that the evidence supporting the efficacy of these medications is very weak.

The diagnostic accuracy of skin tests to Azithromycin, Hydroxychloroquine, and Tocilizumab [22, 31] is low, and to Remdesivir is unknown [32]. A confident diagnosis of drug hypersensitivity can be reached by performing a drug provocation test (DPT) with the culprit drug. Anaphylaxis or severe cutaneous adverse reactions (SCARs) represent a contraindication to DPT with the culprit. So, children with a history of severe reaction to the drugs should undergo skin tests when DPT is contra-indicate. In all the other cases, a DPT should be promptly performed in a hospital setting by qualified personnel with emergency facilities available to treat any reaction. In those cases, with confirmatory diagnosis and an absolute indication of treatment, a desensitization protocol can be settled down with the culprit.

Both immediate and delayed hypersensitivity reactions have been described for macrolides with a prevalence of 0.07–0.7% in children [33]. In the case of Azithromycin hypersensitivity, an antibiotic of the same class (i.e., clarithromycin) can be safely used because of the low grade of cross-reactivity after ascertaining tolerance under medical surveillance. Hydroxychloroquine exposes some patients to the risk of rare but potentially fatal SCARs. Azithromycin and Hydroxychloroquine co-treatment can be safely performed after a cardiological evaluation due to the risks of QT prolongation. Tocilizumab is mostly associated with a local reaction in the injection site, but it has been reported a prevalence of 3.9% of moderate-severe systemic reactions [31].

Heparins [34], mainly low molecular weight heparins, have been widely prescribed as an anticoagulant drug to treat complications of COVID-19 infection. The most common type of hypersensitivity reactions to low molecular weight heparins consists of the local delayed-type ones that may become widespread and occur in 7.5% of patients. Baboon syndrome, DRESS (drug rash occurring with eosinophilia and systemic symptoms) syndrome, AGEP (acute generalized exanthematous pustulosis), and TEN (toxic epidermal necrolysis), are very uncommon. Immediate-type hypersensitivity reactions occur only sporadically. Cross-reactivity of different low molecular weight heparins is a common and unpredictable problem. Allergy tests can be used to identify safe alternative heparin.

In patients with assessed chlorhexidine hypersensitivity, the use of this disinfectant is contraindicated during the COVID-19 pandemic. Low concentrations of chlorhexidine should be used to prevent sensitization.

Chlorhexidine is used as disinfectant and antiseptic, but it is indicated only for children older than 2 months. Allergic reactions are triggered by skin, mucous membrane (oral, conjunctiva, transurethral, rectal, vaginal), and parenteral exposure [35]. Contact delayed reactions like dermatitis can be provoked by its frequent use [35]. Moreover, chlorhexidine can cause immediate systemic reactions such as anaphylaxis [36]. Higher concentrations of chlorhexidine irritate the skin and increase the risk of sensitization [37]. Given the higher use of disinfectants by health workers during the COVID-19 pandemic, an increased incidence of Chlorhexidine allergy could develop. The use of low concentrations of chlorhexidine (0.5–2% weight/volume) is suggested to prevent sensitization.

Patients with latex allergy cannot use latex gloves. Caution is also advised in patients at risk of developing latex sensitization: children with atopic dermatitis, children with any other allergic disease, spina bifida patients, or other conditions with high exposure to latex products in early age. In all these cases, the use of latex-free gloves (i.e., PVC gloves) is suggested.

The use of disposable gloves is suggested as personal protective equipment during the COVID-19 epidemic. Latex hypersensitivity can cause mild cutaneous or respiratory reactions to severe systemic reactions [12]. Latex allergy prevalence ranges from 1 to 5% in the general population. Subjects at risk for latex sensitization include children with atopic dermatitis, children with any other allergic disease, patients with spina bifida and genitourinary malformations, or other conditions with high exposure to latex products in early age. There is a risk also when hypoallergenic latex gloves are used since it means that the additive content is low.

### Urticaria

In patients with chronic spontaneous urticaria (CSU) unresponsive to 2nd generation H1-antihistamines, ongoing treatment with omalizumab should not be discontinued during the COVID-19 pandemic.

Omalizumab, the first available humanized monoclonal anti-IgE in the pediatric population, is recommended as a treatment for children older than 12 years with chronic spontaneous urticaria (CSU) unresponsive to 2nd-generation H1-antihistamines [38]. This therapy is recommended to be administered as a subcutaneous injection at 300 mg every 4 weeks [1]. During COVID-19 pandemic and consistently with health conditions, patients with CSU should be advised to continue to use their routine treatment, including omalizumab, to prevent disease exacerbations, unnecessary visits to physicians, and hospitalization [39, 40]. In this regard, patients can be trained to self-inject omalizumab and stay at home if they are competent and confident enough [5, 6]. Follow-up appointments should be scheduled if needed. New patients should receive the first two injections of omalizumab in the hospital due to the minor risk of anaphylaxis; then, they can continue treatment at home [39, 40]. Delaying the beginning of omalizumab treatment is not suggested as giving long-term high corticosteroid doses or using other immunosuppressive drugs could increase the vulnerability to COVID-19 [40].

In patients with CSU unresponsive to 2nd generation H1-antihistamines and omalizumab, ongoing treatment with cyclosporine A should not be stopped during the COVID-19 pandemic.

Cyclosporine A has shown an excellent clinical efficacy when administered in combination with 2nd-generation H1-antihistamine in treating children with CSU. However, due to a higher incidence of adverse effects, cyclosporine A is not recommended as a standard treatment in CSU. On the other hand, in children and adolescents with CSU unresponsive to 2nd generation H1-antihistamines and omalizumab, cyclosporine A is suggested as a therapeutic option [38]. Similar to patients undergoing immunosuppressive treatment, cyclosporine A should not be stopped during COVID-19 pandemic and consistently with health conditions; however, the administration of the lowest possible doses (≤1 mg/kg/day) is suggested due to a minor increased risk of viral infections, mainly respiratory tract infections [39, 41]. For patients with a new CSU diagnosis unresponsive to 2nd generation H1-antihistamines and omalizumab, we suggest delaying the beginning of cyclosporine A treatment consistently with health conditions.

Ongoing treatment with immunosuppressive and/or biologic agents should be discontinued in patients with CSU who have tested positive for COVID-19 or with suspected COVID-19 infection.

The use of immunosuppressive agents, such as cyclosporine, for dermatologic diseases is contraindicated in patients with active infections [42]. Since available data suggest multiple cytokine axis activations in the viral immune response in patients with COVID-19, broad immunosuppression may have the potential to increase susceptibility, persistence, and reactivation of the viral infection. Immunosuppressive agents may decrease cytokines that recruit and differentiate immune cells needed to clear the infection. Also, inflammatory mediators may become hyperactivated, resulting in a “cytokine storm,” which has been demonstrated to be involved in severe and life-threatening COVID-19 [43]. Biologic agents are targeted therapies that tend to be less involved in the previously mentioned components of the viral immune response. Whether biologic agents may increase the risk of infection or cause a severe disease is unknown.

### Rare allergic diseases

In pediatric patients, with a history of systemic reactions to Hymenoptera venom not isolated to the skin, a timely evaluation should be carried out on time. Moreover, an adrenaline auto-injector should be promptly prescribed, and venom immunotherapy (VIT) should be punctually started if deemed required after an appropriate diagnostic workup. VIT should be continued during the COVID-19 pandemic in pediatric patients who are currently undergoing the treatment. According to an individual cost-benefit ratio, spacing the VIT doses may be considered in patients in the maintenance phase.

Systemic reactions to Hymenoptera venom could be life-threatening [44–46]. Thus, the appropriate diagnostic workup, the prescription of an adrenaline auto-injector, and VIT’s performance are critical services, and they should not be delayed or suspended.

In patients with suggestive symptoms of eosinophilic esophagitis (EoE), the esophagogastroduodenoscopy should not be deferred in case of severe dysphagia or dyspepsia with alarm symptoms (i.e., weight loss). The esophagogastroduodenoscopy is mandatory in case of food impaction. In patients on proton pump inhibitor therapy, in clinical remission, it is advised to suspend the therapy progressively. In case the symptoms recur, a cycle of swallowed topical steroid, supported by pediatricians/allergist via telemedicine, if possible. In patients on swallowed topical steroid therapy is recommended to continue with the minimum effective dose. Food challenge for the reintroduction of avoided foods should be delayed or suggested via telemedicine, if possible.

Many patients with EoE are generally healthy except for atopic comorbidities such as asthma, allergic rhinitis, eczema, and IgE mediated food allergy, which must be maintained under control [47, 48]. Most of the care for patients with EoE could be handled via telemedicine, except for patients with food impaction that required urgent endoscopy procedures or dysphagia or dyspepsia with alarm symptoms endoscopy should not be deferred.

### Immunization and immunodeficiency

Routine immunization activities, both for primary cycles and boosters, as per National vaccine schedule recommendations, have to be maintained unless the Referral Center reports specific contraindications for the child with allergy and/or immunodeficiency. This recommendation also applies to household members.

Immunization represents a priority public health service in the era of COVID-19 pandemic to prevent vaccine-preventable diseases (VPDs) for the benefit of the entire community.

Vaccine-preventable diseases could reemerge if vaccinations are not routinely administered due to the COVID-19 pandemic, with a dramatic impact on the Health System [49, 50]. Therefore, routine immunization activities, both for primary cycles and boosters, as per national vaccine schedule recommendations, have to be maintained, unless specific contraindications for the child with allergy and/or immunodeficiency exist [51]. Protection measures for children and healthcare workers should be used.

When routine immunization activities are not ensured because of the burden of COVID-19 on the health care system, the immunization service providers should create cohorts of children who have missed their vaccine doses and activate plans of recalling for catch-up immunization [49, 50].

It is desirable, whenever possible, to coadminister vaccines during the same visit (e.g., MenB vaccine with the hexavalent vaccine or anti-C/ACWY MenC; Meningococcal ACWY vaccine with MMRV).

Reduce the burden of immunization services, the coadministration of vaccines during the same visit is strongly recommended [49, 50].

Routine influenza immunization should be considered in all children during the 2020–2021 season to ensure protection measures for children and healthcare operators. Inactivated influenza vaccine should be likewise considered in all primary immunodeficiency (PID) patients unless medical contraindications exist or the patient is considered unable to mount an effective immunological response. Nonviable influenza vaccination is also strongly recommended in household contacts of PID patients.

The American Academy of Pediatrics, unless specific contraindications exist, recommended routine influenza immunization in all children starting at 6 months of age during the 2019–2020 season with greater emphasis in high-risk groups [52]. Clinical spectrum of COVID-19 and influenza infections are very similar [53]. Nevertheless, some primary immunodeficiency (PID) patients, especially those with chronic lung disease, might have a more severe clinical course [54]. During the COVID-19 pandemic, reinforcement of influenza immunization programs has also been suggested for 2020–2021 by the Italian Pediatric Society, with particular consideration for children under 6 years of age and patients with chronic underlying conditions [50]. The use of airborne precautions in the office for both children and healthcare operators is recommended during immunization procedures. Annual immunization with a nonviable influenza vaccine is also recommended in all PID patients if the patient is considered to be capable of mounting an effective immunological response unless medical contraindications exist [51]. Nonviable influenza vaccination is also strongly recommended in household contacts of PID patients.

Immunoglobulin replacement therapy represents a life-saving treatment for many primary immunodeficiencies (PIDs), including hypo/agammaglobulinemia and immunodeficiencies characterized by combined or complex phenotypes [54–56]. SARS-CoV-2 RNA has been detected in plasma or serum collected from symptomatic COVID-19 patients but also in asymptomatic blood donors [57, 58]. Nevertheless, the transmission of SARS-CoV-2 through blood products so far has not been established [53, 58, 59]. The safety of immunoglobulins against virus contamination is also ensured through multiple virus inactivation and removal steps during manufacturing processes [54]. Therefore, immunoglobulin replacement therapy should be continued in PID patients, without changing the timing and the dose of administration unless clinically indicated. The use of home services (IV/SC) is therefore encouraged, provided that proper training procedures have been ensured [58].

In PID patients, investigation for SARS-CoV-2 is recommended after public exposure and when acute respiratory or gastrointestinal symptoms occur. After exposure to SARS-CoV-2 in PID patients, the risk/benefits of continuing immunosuppressive or immunomodulatory therapy should be discussed with the immunology specialist.

Some PID patients, especially those associated with chronic lung disease, might be at higher risk for a more severe course of COVID-19 [60, 61]. Stratification of risk has also been proposed by some immunological societies [62]. However, very few data exist on the clinical course and phenotype of COVID-19 in PIDs [58]. It is also conceivable that some immunosuppressive therapies (e.g., corticosteroids) can attenuate some clinical symptoms [54, 56]. Investigation for SARS-CoV-2 is recommended after public exposure in severe forms of PIDs and when acute respiratory or gastrointestinal symptoms occur, according to also to health authorities guidelines [11]. It must be remembered that in some patients, the viral shedding could be prolonged because of a slow clearance [54]. After SARS-CoV-2 exposure, the decision to continue immunosuppressive or immunomodulatory therapies should be discussed with the immunologist [54, 59]. Drug interactions must also be carefully considered.

According to also the phenotype and severity of COVID-19 disease, these PID patients should have easy access to disposable face masks and other personal protective equipment. Their use should be made according to health authorities’ provisions and the best information obtained from scientific reports and international guidelines.

Many PID patients and their households, according to also to the phenotype and severity of the disease, should have easier access to disposable face masks and other personal protective equipment. However, it is unlikely that airborne precautions could be sufficient if not supported by careful and frequent hand washing, social distancing, and other general preventive measures [58, 59, 63]. Protective equipment should be used according to standardized procedures, according to also to health authorities provisions and the best information obtained from scientific reports and international guidelines.

Very recently, three Italian studies concerning this issue have been published [64–66].

### Allergen immunotherapy

Both subcutaneous (SCIT) and sublingual (SLIT) immunotherapy can be continued during the current COVID-19 pandemic, in all asymptomatic patients, in healthy children with negative test results, without exposure or recent contact with subjects symptomatic or virus-positive or who have not recently traveled to high-risk areas. In particular, immunotherapy for Hymenoptera venom (SCIT for bee, wasp, and bumblebee) must be regularly administered, is a life-saving drug. Venom immunotherapy is a life-saving treatment to prevent anaphylaxis, as is effective in 100% of the subjects treated [67]. Venom therapy could avoid single-use of adrenaline.

Stopping subcutaneous immunotherapy during the COVID-19 pandemic is not advisable [67]. The possibility of increasing the duration of the intervals between the administration in the maintenance phase must be assessed on a case-by-case basis and can be useful when we do not want to expose the child or the family to an inconvenient transfer to reach our clinic or in case of recent contact with infected people. The administration of SLIT to the child or adolescent at home makes it very advantageous to avoid contact with potentially infected people [67]. Interrupting SLIT is never recommended, except in this case, there have been recent contacts with infected people or even mild, but suggestive symptoms of viral infection are present. Clinically, these results are manifested in better control of the allergic rhinitis symptoms and, especially, in asthma prevention, which could be confused with symptoms of viral infection. Moreover, AIT determines a decrease in the medication use (corticosteroids and inhaled therapy) that could reduce the risk of spreading viral infections (like COVID-19) in other subjects and the possible worsening of a viral infection in the patient himself.

Immunotherapy (SCIT and SLIT) must be interrupted immediately:

- in symptomatic patients exposed to contact with COVID-19 positive individuals
- in asymptomatic patients, but positive test results
- in patients with an accentuation of respiratory symptoms related to possible virosis, even if only an allergic component is suspected.

It is considered dangerous to continue AIT in individuals with suspected or confirmed infection with COVID-19 [67]. Moreover, it is useful to avoid confounding effects such as the accentuation of symptoms in subjects with seasonal symptoms (allergic rhinitis and/or asthma episodes), also following the administration of immunotherapy and possible concomitant viral infections. In this case, it would be more complex to effectively distinguish the clinical manifestations, viral and/or allergic, often present concomitantly and which could delay an adequate diagnosis and treatment of the COVID-19 infection.

### Aerobiology and environment

The extensive use of personal protective equipment represents the primary defense for allergic children during a COVID-19 pandemic.

Face masks, even the self-produced ones, are an indispensable safeguard in community activities’ resumption, protecting bi-directionally from droplets [68–70]. The filterless FFP2 / 3 respirators represent an optimal primary and bi-directional defense even against pollen, mites, and viruses. It is essential to avoid that the rhinitis symptoms related to pollinosis can be confused with the COVID-19 infection, generating situations of concern. The correct size of the mask is fundamental because it affects the fit on the face and its effectiveness.

#### Promote periodic ventilation, both in domestic and indoor community environments

The size of the SARS-CoV-2 virus is within a range of 50–200 nm, falling within the nanoparticle range, which, especially in indoor environments, can reach high degrees of diffusion [71–73]. Indeed, the SARS-CoV-2 virus produced an aerosol from expectoration, respiration, and vocalization that can remain in the atmosphere for a period of between 3 and 16 h as bioaerosol, constituting a potential danger. Therefore, extensive ventilation in domestic and community environments in the allergic child could help in reducing exposure to indoor allergens and SARS-Cov-2 as well [74, 75].

Reduce air pollutants in the outdoor environment and promote “gentle mobility.”

Outdoor pollution might have a role in COVID-19 incidence and severity [76]. Therefore, reducing particulate matter (PM_2.5_ and PM10) in the atmosphere, paying great attention to the problem of the vehicle and industrial emissions, could promote not only respiratory health but also children less vulnerable to SARS-CoV-2 [77, 78].

### Practical problems

Outpatient clinic allergology consultation and skin prick test (SPT) in children with suspect respiratory allergy and/or food allergy [79].

Allergology consultation and SPT in children with the specific 2019-nCoV infection must not be performed.

Allergology consultation and SPT in children with suspected 2019-nCoV infection (e.g., cough, rhinitis, fever, and epidemiological criteria) must not be performed.

If the child and/or the accompanying parent’s infectious status is unknown regarding 2019-nCoV, it is necessary:

1. Space appointment to avoid contact between the previous patient and the next, ventilate the room between visits if a negative pressure room is not available, sanitize the surface where the child’s forearm was placed to perform the SPT;
2. The health care provider, the patient and the accompanying parent must wear certified protective masks (preferably FFP2/N95) and must wash their hands immediately before the visit;
3. Distance between the health care provider, the child and the accompanying parent must be more than 1 m;
4. Sanitize medical tools used in different patients.

### Diagnostics

Allergy diagnostic procedures during the COVID-19 pandemic should be delayed, unless the workup is essential for safety reasons, in the allergic patient [2].

So far, scientific societies and researchers have not expressed real recommendations concerning what kind of allergic procedure should be assured during the COVID-19 pandemic. Most of the recommendations here listed are therefore issued from the experts’ opinion. Specialists should evaluate the risk/benefit ratio in each patient to decide which tests should not be delayed, even during the pandemic context, still trying to reduce as much as possible the risk of spreading the viral infection. In general, not deferrable clinical patterns requiring a fast allergy workup should include:

- a recent history of anaphylaxis, with a need, for the child, to diagnose the allergen responsible of the clinical picture (mainly for food and drug allergies) and to provide a therapeutic plan and self-injectable adrenaline;
- the appearance of symptoms evocative of food protein-induced enterocolitis syndrome (FPIES), if the culprit food is essential from a nutritional point of view for the toddler, and to avoid the further appearance of acute symptoms;
- IgE mediated food allergies, such as cow’s milk allergy, wheat allergy, or allergies to other nutritionally essential food;
- suspected drug allergies, in patients who need treatment with the possible culprit drug (including antibiotics in cystic fibrosis patients, patients with tuberculosis or immunocompromised; vaccines in immunocompromised children; chemotherapeutic agents in oncologic patients);
- severe eczema, especially in cases of large areas of the body are interested, or a history of cutaneous infection;
- severe reactions to Hymenoptera venoms, which are more frequent during spring and summer seasons.

Allergy diagnostic procedures should be performed in safety settings, with minimal exposure of the children, the caregiver, and the medical staff.

Diagnostic procedures should be performed in a safe setting, with the presence of one parent only for each child, and with the medical staff properly equipped to reduce the risk of infection for the staff, the child, and the caregiver.

In those cases, in which a diagnostic workup is needed, in vitro tests should be preferred, such as specific IgE, rather than skin prick tests. Drug challenges to diagnose an allergy or to confirm a hypersensitivity reaction, or to explore possible alternatives (antibiotics, NSAIDs, perioperative agents) are also acceptable in specific patients.

### Social consequences of lockdown

The COVID-19 pandemic has diverted all resources to its management and imposed drastic containing measures, such as the suspension of school activities. The effects on patients suffering from allergic respiratory pathology are twofold. On the one hand, the difficulty in accessing the pediatric allergology offices has resulted in the interruption of direct contact between patient, family, and specialist. On the other hand, the virus led to the sheer anxiety and fears of both patients and their families. In this scenario, “Therapeutic Education” is of crucial importance. This approach implies that families and specialists work together towards a planned and organized transfer of therapeutic skills. The aim is to equip patients and families with practical knowledge in the absence of scheduled visits. As a result, they will feel less alone and more prepared to deal with disease management. Telemedicine may provide a fundamental contribution to maintain remote contact, although access to technology remains challenging for the most fragile social groups [80].

Parents should be encouraged to continue specific therapies even during COVID-19 infections:

- Corticosteroid drugs for topical or oral use, short- or long-acting bronchodilators by inhalation spray or powder, anti-leukotriene agents, biological drugs, intramuscular adrenaline should be administered according to the individual therapeutic plan prescribed. There is no evidence that these therapies may suppress immunity or induce deterioration in case of COVID-19 infection;
- oral antihistamines should be continued regularly to keep the skin, eyes and nasal allergy symptoms well controlled from allergens exposure;
- for subjects not infected (or cured) by COVID-19 in subcutaneous or sublingual immunotherapy (SCIT or SLIT), the therapeutic plan must not be interrupted. SLIT is administered at home, has the advantage of avoiding contact with people infected. SCIT or SLIT must be suspended in case of a positive test or suspicion thereof immediately upon the appearance of symptoms of COVID-19 infection (or potential symptoms);
- active and passive cigarette smoking is not recommended;
- in the event of further deterioration, contact the doctor and/or emergency lines, such as 112. Direct access to the emergency room must be limited only for extreme urgency and seriousness [81].

When schools open again, allergic children must be guaranteed:

- social distancing, washing hands frequently with warm running water with ordinary soap (to be rubbed on all areas of the hands for at least 30 s) or with disinfectant gels without rinsing. To be noted that washing hands should not be excessive in children with atopic dermatitis.
- for children with a food allergy, the exclusion of the food in question;
- emergency therapy, self-injectable intramuscular adrenaline, and salbutamol spray, according to the individual therapeutic plan [82].

During a COVID-19 pandemic, asymptomatic allergic children have the right to undergo all recommended childhood vaccinations. Failure to comply with the vaccination calendar can frustrate the immunization, which is necessary for the child’s defenses and herd immunity, and facilitate high-risks situations, such as possible epidemic outbreaks [83].

### Telemedicine

In the management of patients with suspected or ascertained allergic or immune-mediated diseases, encourage remote telehealth medicine, not deferring hospital-based consultations when red-flags for an urgent visit exist [84].

In the COVID-19 era, telehealth medicine could play a key role in delivering immunological and allergological consultations and ensuring the need to keep social distancing. Activating virtual consulting practices by the opportunities offered from free access web-based communication platforms may be beneficial for both first consultations and follow-up visits in patients with suspected or ascertained allergy or immunodeficiency. Sharing between families and allergy or immunology specialists clinical materials, such as images, investigations, or laboratory data, may help manage many simple immune-mediated diseases like urticaria or recurrent respiratory infections. Indeed, history plays a pivotal role in establishing when parents’ perception of hypersensitivity or immune-mediated symptoms fit with a defined allergic or immunodeficiency condition or an exacerbation of a previously diagnosed immunological disease occur. At the same time, specialists must not overlook some crucial symptoms when an overt emergency occurs. As an example, in the table (Table 1) are reported some “red flags” which could help the clinician in establishing if a child with the suspected allergic or immune-mediated disease need for an urgent hospital-based consultation, with the possible hypothesis of symptom-related underlying clinical conditions.

**Table 1:** Example of clinical symptoms and related laboratory or instrumental findings indicating the need for an allergy or immunology specialist’s need for an urgent consultation

Presenting symptoms and/or laboratory findings	More probable allergic and/or immune-mediated disease	Possible alternative diagnosis
Uncontrolled asthma-like symptoms	Severe asthma or asthma with comorbidities (asthma plus)	Dysfunctional breathing disorders, aspiration disease, foreign bodies (< 6 yr), ANCA-associated pulmonary vasculitis, interstitial lung disease
Recent severe asthma attack	Uncontrolled asthma	Dysfunctional breathing disorders, aspiration disease, foreign bodies (< 6 yr)
Chronic diarrhea with failure to thrive and/or malabsorption	FPIES or eosinophilic gastroenteritis	Combined immunodeficiency, cystic fibrosis, autoimmune enteritis, gastrointestinal infections
Acute hypersensitivity symptoms occurring after the first introduction/s of a food^a^	Anaphylaxis	FPIES, spontaneous urticaria
Acute hypersensitivity symptoms occurring after drug administration^b^	Anaphylaxis	Mastocytosis or idiopathic mast cell activation syndrome, spontaneous urticaria
Severe cutaneous adverse reaction (SCAR) w/wt systemic symptoms occurring after drug administration^b^	DRESS, TEN, Stevens-Johnson syndrome	viral infection (EBV, CMV, HHSV6, others), staphylococcal and streptococcal shock syndrome, autoimmune diseases
Acute hypersensitivity symptoms occurring after vaccine administration ^c^	Anaphylaxis (rare)	Spontaneous urticaria
Chronic unexplained cough	Wet: protracted bacterial bronchitis, bronchiectasis Dry or mixed: foreign body, pertussis or parapertussis, or Mycoplasma infection	Pulmonary tuberculosis, tracheobronchomalacia, vascular rings/slings, aspiration syndrome, cystic fibrosis, primary ciliary dyskinesia
Prolonged respiratory symptoms and/or interstitial pneumonia, especially if combined with poor weight gain	Combined immunodeficiency	interstitial lung disease, cystic fibrosis, Shwachman disease
Hypereosinophilia (not allergy-related)	Hypereosinophilic syndromes, parasitic disease	Malignancies, primary atopic disorder
Severe neutropenia (< 0.5 × 10^9^/L) in the context of a pyogenic infection	Severe congenital neutropenia (SCN)	Drug-related agranulocytosis, aplastic anemia, autoimmune neutropenia (rare)
Severe lymphopenia in the context of severe or atypical infections and/or failure to thrive	Severe combined immunodeficiency (SCID)	Other primary immunodeficiencies
Recurrent fever in a “sick” appearing child	Autoinflammatory disease	Malignancies, chronic inflammatory bowel disease, recurrent organ infections in an immunocompromised child
Multiple autoimmune diseases	Primary immunodeficiencies, systemic rheumatological disease	

^a^the condition needs urgent consultation if the suspected food is not easily replaceable in the child’s diet (e.g., cow’s milk)

^b^ the condition needs urgent consultation if the suspected drug is not replaceable in a specific clinical condition (e.g., cystic fibrosis, neoplasms)

^c^ the condition needs urgent consultation if primary series vaccination are interrupted

## Conclusions

A recent meta-analysis investigated the impact of COVID-19 on the pediatric population [85]. A total of 815 articles were identified. Eighteen studies with 1065 participants (444 patients were younger than 10 years, and 553 were aged 10 to 19 years) with confirmed SARS-CoV-2 infection were included in the final analysis. All articles reflected research performed in China, except for one clinical case in Singapore. Children at any age were mostly reported to have mild respiratory symptoms, namely fever, dry cough, and fatigue, or were asymptomatic. Bronchial thickening and ground-glass opacities were the main radiologic features, and these findings were also reported in asymptomatic patients. Among the included articles, there was only 1 case of severe COVID-19 infection, which occurred in a 13-month-old infant. No deaths were reported in children aged 0 to 9 years. Available data about therapies were limited. More recently, an Italian report demonstrated that children and adolescents rarely suffered from COVID-19, and most of them required no or low-intensity care [86]. Another study reported that adolescents were influenced by COVID-19 concerning emotional aspects [87].

Therefore, COVID-19 seems to affect childhood and adolescence scarcely, as very recently demonstrated in Italy [88]. However, allergic and immunodeficient children and adolescents need adequate care in this period. Treatments should not be interrupted, and telemedicine should be implemented. The current Consensus Statement could provide timely and updated information for pediatricians and caregivers.

On the other hand, a radical re-organization of the healthcare system should be considered, considering the local peculiarity, such as infection-free areas, medical staff replacement for a long time, cost sustainability, and medical education. An extensive debate and appropriate measures should be considered.

## Data Availability

Not applicable.

## Abbreviations

SARS-COV-2: Severe acute respiratory syndrome – coronavirus
COVID-19: Coronavirus disease 2019
SIAIP: Società Italiana di Allergologia ed. Immunologia Pediatrica
ICS: Inhaled corticosteroids
OCS: Oral corticosteroids
MDI: Metered dosed inhaler
PPE: Personal protective equipment
ACE2: Angiotensine converting enzyme 2
FA: Food allergy
OFC: Oral food challenge
OIT: Oral immunotherapy
WHO: World Health Organization
AD: Atopic dermatitis
NICE: National Institute for Health and Care Excellence
DPT: Drug provocation test
SCAR: Severe cutaneous adverse reactions
CSU: Chronic spontaneous urticaria
VIT: Venom immunotherapy
PID: Primary immunodeficiencies
SLIT: Sublingual immunotherapy
SCIT: Subcutaneous immunotherapy
PM: Particulate matter
